# Gastrointestinal stromal tumor (GIST) presenting as a multilocular cystic intra-abdominal mass in a dog

**DOI:** 10.1186/s12917-022-03504-0

**Published:** 2022-11-26

**Authors:** John Edward Blaxill, Hannah Bender, Qicai Jason Hoon, Jia Wen Sow, Katrina Y. Cheng, Peter Francis Bennett

**Affiliations:** 1 University of Sydney Veterinary Teaching Hospital, Evelyn Williams Building B10, 65 Parramatta Road, Camperdown, New South Wales 2050 Australia; 2 Vetnostics, 60 Waterloo Rd, North Ryde, New South Wales 2113 Australia; 3 Surgery Department, Veterinary Specialist Services (Underwood), 1-15 Lexington Road, Underwood, Queensland 4119 Australia; 4 Department of Diagnostic Imaging, Small Animal Specialist Hospital (SASH), Level 1, 1 Richardson Place, North Ryde, New South Wales 2113 Australia

**Keywords:** Mesenchymal, Neoplasia, Abdominal, Peritoneal, c-Kit, CD117, Toceranib, Cyst

## Abstract

**Background:**

Gastrointestinal stromal tumor (GIST) is a malignant mesenchymal neoplasm described in humans, dogs, and cats. A hallmark of diagnosis for GISTs is positive immunohistochemical labelling with *c-Kit* (CD117). The differentiation of GIST from other mesenchymal neoplasms of the gastrointestinal tract is pivotal to allow for initiation of appropriate treatment. In humans, cystic GIST has been described, though this has not been reported in dogs. In humans, the cystic form of GIST has been associated with a favorable prognosis. In the present paper, we report a case of multilocular cystic GIST in a dog, which has not previously been described in this species.

**Case presentation:**

A ten-year-old, male-entire Maltese terrier mix breed dog presented with a large cystic mural mass of the duoedenum and orad jejunum. Histopathology and positive immunohistochemical staining with CD117 confirmed a diagnosis of GIST. No evidence of metastasis was detected on routine staging with abdominal sonography and thoracic radiography at the time of diagnosis. Surgical resection was performed and toceranib therapy was initiated post-operatively. Metastasis was documented 251 days after surgery on computed tomography. Due to clinical deterioration, the patient was humanely euthanised 370 days after surgical excision.

**Conclusions:**

There are few differential diagnoses for large multilocular cystic intra-abdominal masses in dogs. This case presents a previously undescribed presentation of gastrointestinal stromal tumor in the dog as a predominantly multilocular cystic mass. It remains unclear if the cystic form of GIST may represent a favorable prognosis in dogs.

## Background

Mesenchymal tumors account for 10 – 30% of neoplasms in the gastrointestinal tract of dogs [1, 2]. The most common of these are leiomyosarcoma and gastrointestinal stromal tumor (GIST), with each accounting for around half of cases reported in dogs [1–3].

GISTs most often occur in the small intestine and cecum of dogs, though in humans these are more common in the stomach [4]. GISTs are typically solid neoplasms, though rare presentations of predominantly cystic neoplasms are reported in humans [5]. Cystic GISTs have not previously been described in the veterinary literature. This report describes a case of GIST with predominant cystic change in a dog.

## Case report

A 10-year-old, male entire, Maltese terrier mix breed dog presented with a three-month history of slowly progressive abdominal distension. The patient was reported to vomit bilious material daily for two weeks prior to presentation and had no other significant clinical history. Physical examination identified abdominal distension with no overtly palpable abdominal mass effect, a grade IV/VI left-sided apical systolic cardiac murmur and generalized moderate muscle atrophy.

**Fig. 1:**
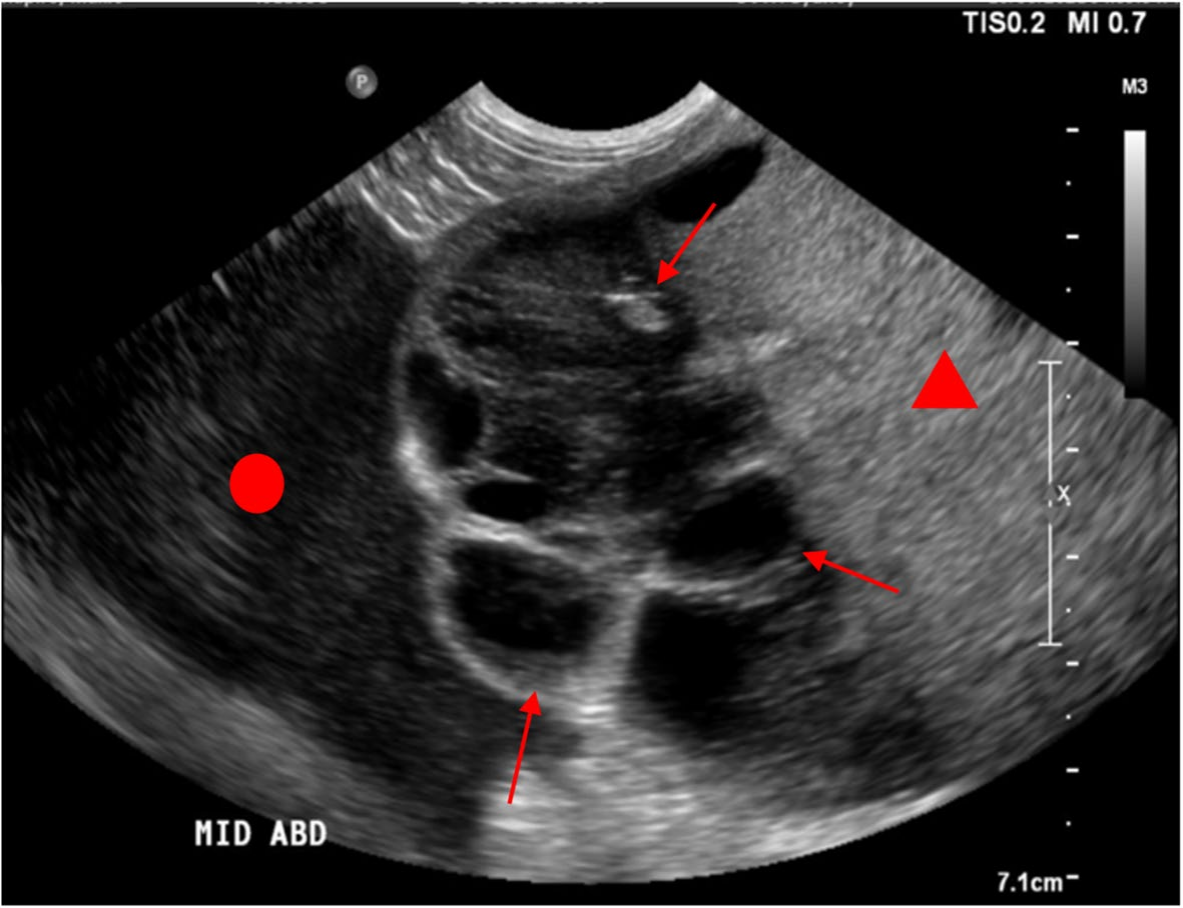
Sonographic image of a portion of the mid-abdominal mass including solid portions (red triangle) and cystic components (red arrows; not all components labelled). In the cranial aspect, there is a triangular echogenic fluid pocket of uncertain origin (red circle) – this may be another cystic component of the mass or free in the peritoneal cavity. Cranial is to the left of the image

Echocardiographic examination identified chronic degenerative mitral valve disease, most consistent with myxomatous mitral valve disease. Sonographic examination of the abdominal cavity identified a markedly expansile, heterogeneously echogenic, well-vascularized soft tissue mass with multiloculated cystic structures containing anechoic fluid admixed with echogenic particulate matter (Fig. 1). The origin of the mass could not be determined. The mass extended cranially to the level of the liver, caudally to the level of the bladder, and dorsally to the level of the kidneys. Other findings included a mild reduction of renal corticomedullary definition bilaterally, diffusely hyperechoic hepatic parenchyma, and a symmetrically enlarged prostate with cystic areas. The remainder of the abdominal sonographic examination was unremarkable. Thoracic radiography identified no abnormalities.

Abdominocentesis removed 700 mL of transparent straw-colored fluid, identified as transudate (protein 26 g L^−1^) with low numbers of large mononuclear cells (120 × 10^6^ cells L^−1^). The cellular population of the abdominal fluid consisted of large mononuclear cells with vacuolated grey to light blue cytoplasm, with a round, irregular medium to large nucleus most likely of monocyte lineage. Complete blood count^1^ and serum biochemical analysis^2^ were unremarkable. Resting ammonia^3^ was within reference range. Prothrombin time and activated partial thromboplastin time^4^ were within normal limits.

**Fig. 2:**
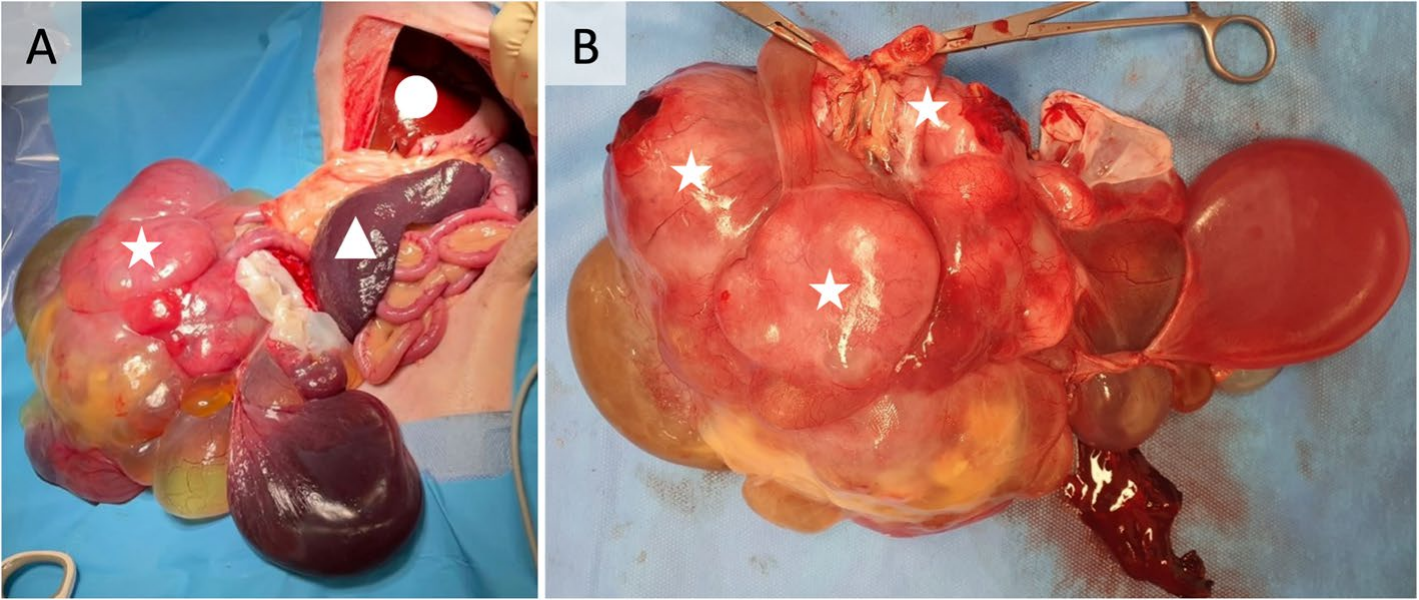
Intraoperative photographs of the cystic gastrointestinal stromal tumor. **A** demonstrates structures including the duodenum and jejunum (star), liver cranially (circle) and spleen (triangle). **B** demonstrates the intermingled relationship of the gastrointestinal tract with the GIST

A multilobulated cystic mass arising from the duodenum and orad portion of the jejunum with omental adhesions was identified during ventral midline celiotomy (Fig. 2). The longest diameter of the mass measured approximately 20 cm. The adhesions were broken down and the mass was resected with 5 cm orad and aborad margins^5^. A functional end-to-end stapled anastomosis of the duodenojejunum was performed. A focal nodule identified within the left lateral liver lobe was biopsied. The remainder of the peritoneal cavity examination was unremarkable.

**Fig. 3:**
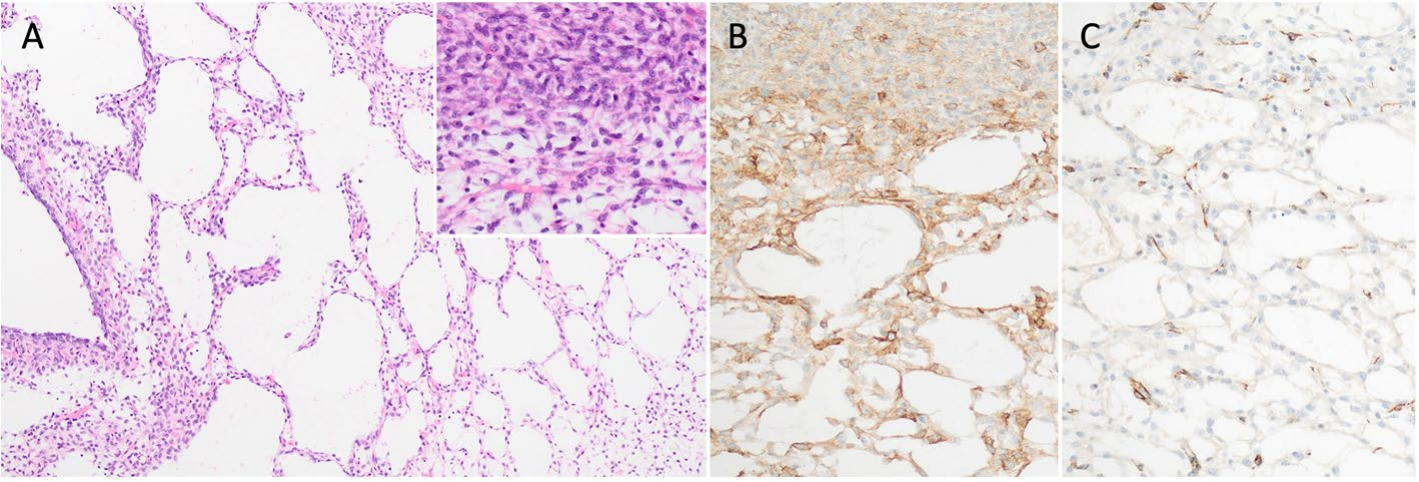
**A** The mass is composed of neoplastic mesenchymal cells arranged into delicate streams that intersect multilocular, empty cystic spaces. Inset: On higher magnification, neoplastic spindle cells have indistinct cell margins, scant vesicular cytoplasm and oval-shaped nuclei with small, centrally located nuclei. **B** IHC for CD117. Moderate membranous labelling is detected in the majority neoplastic cells. **C** IHC for smooth muscle actin. Light cytoplasmic labelling is present in rare neoplastic cells

Histopathologic examination identified an unencapsulated, cavitated mass composed of monomorphic mesenchymal cells arranged in interlacing streams and loosely organised herringbone patterns with variable collagenous or myxoid matrix. Empty cystic spaces multifocally scattered throughout the mass were not lined by epithelial cells (Fig. 3A). Neoplastic cells were characterised by indistinct margins, small amounts of vacuolated, amphophilic cytoplasm and oval-shaped nuclei with marginated chromatin and small, centrally located nuclei. Anisocytosis and anisokaryosis were mild, and the mitotic count was five per 10 high power fields (equivalent to 2.37mm^2^). A preliminary diagnosis of nonangiogenic, nonlymphogenic intestinal mesenchymal tumor (NIMT) was made based on histopathology alone. NIMTs are a subset of poorly defined intestinal sarcomas that require immunohistochemistry (IHC) for further classification between the primary differential diagnoses of GIST and leiomyosarcoma. In this case, IHC for CD117 and smooth muscle actin were required to distinguish between primary differential diagnoses of GIST and leiomyosarcoma, and exclude less likely differentials including fibroblastic and neural tumors.

IHC for CD117 demonstrated moderate-to-strong membranous labelling of greater than 95% of neoplastic cells (Fig. 3B). IHC for smooth muscle actin yielded variable cytoplasmic staining among neoplastic cells (Fig. 3C). GIST was diagnosed based on positive immunolabelling with CD117.

Hepatic biopsies identified no metastatic disease. Mild hepatocellular glycogen accumulation and lipogranuloma formation, considered incidental findings, accounted for the gross appearance of the liver during surgery. Hepatic biopsy specimens were submitted for bacterial culture and sensitivity due to pale-yellow discoloration of the liver at time of surgery. Aerobic and anaerobic bacterial culture of hepatic tissue identified no bacterial growth.

The patient was started on toceranib^6^ at 3.0 mg kg^−1^ orally three times per week on a Monday, Wednesday, and Friday dosing schedule. No local disease recurrence or metastasis was noted on examination 78 days after starting toceranib.

**Fig. 4:**
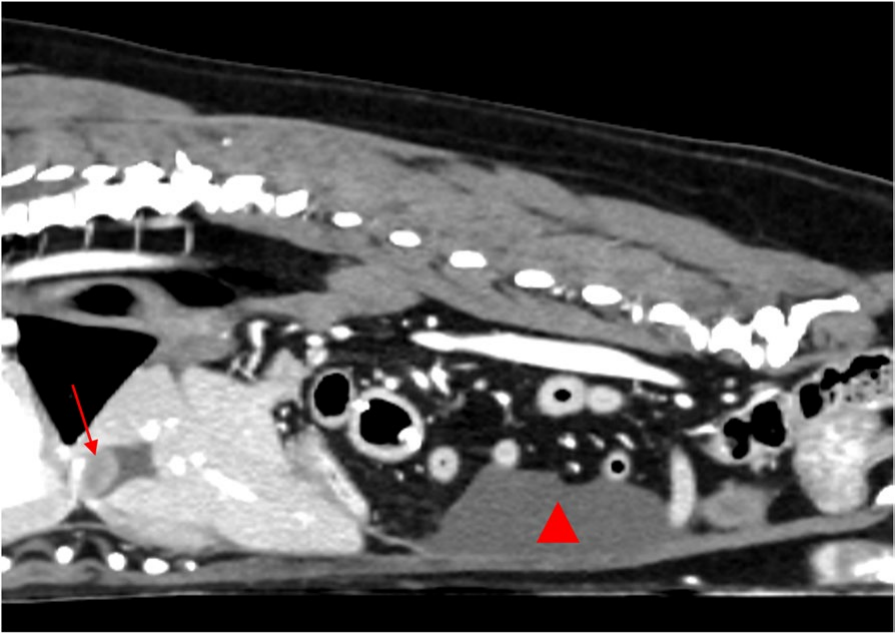
Sagittal plane computed tomographic image identifying an abnormal cystic structure of the cranial abdomen (red arrow) and encapsulated peritoneal effusion (red triangle) at time of suspected disease progression (metastasis)

Metastasis of GIST was strongly suspected at recheck examination 251 days after surgery based on computed tomographic imaging, which identified multiple peritoneal nodular changes and a small volume of encapsulated peritoneal effusion (Fig. 4). No evidence of pulmonary metastasis, and no local recurrence at the duodenojejunal enterectomy site was identified on computed tomography. Toceranib was discontinued approximately two months prior to this recheck examination and diagnostic imaging study due to owner non-compliance; the patient had a one-month history of mild lethargy prior to the recheck. Peritoneal fluid analysis identified a macrophagic exudate without evidence of neoplastic cells. No specific sampling of the suspected metastatic lesions was performed due to an inability to access these sites safely.

The patient was re-initiated on toceranib at 3.0 mg kg^−1^ three times per week on a Monday, Wednesday, and Friday dosing regimen at the time of suspected metastasis. There was initial improvement in clinical signs of lethargy after re-initiation of therapy. The patient later presented for worsening lethargy and anorexia of one week duration, and owners elected for humane euthanasia due to a poor prognosis. The overall survival time was 370 days. Post-mortem examination was declined by the owners.

## Discussion and conclusion

This report highlights an important differential diagnosis for a predominantly cystic mass within the abdominal cavity of dogs. Differential diagnoses for dogs with intra-abdominal cystic lesions include cystic lymph nodes, congenital duplication cysts, pancreatic pseudocyst, paraprostatic cysts, cystic lesions of the female reproductive tract, parasitic disease, and cystic neoplastic disease. These conditions are generally localized, whereas this case was expansile without clear lesion localisation prior to exploratory celiotomy.

It is speculated that GISTs arise from the interstitial cells of Cajal, which are pivotal to coordination and propagation of intestinal slow waves and allow transduction of neural inputs from the enteric nervous system [6]. These are generally solid neoplasms of spindle cells arising from the gastrointestinal tract of dogs which can be differentiated immunohistochemically from other intestinal mesenchymal neoplasms by positive expression of the type III tyrosine-protein kinase *c-Kit* (CD117) and discovered-on-GIST-1 (DOG-1) [2, 3, 7].

In humans, it has been speculated that cystic lesions of GISTs may occur due to (a) rapidly expansile growth pattern in which cystic structures occupy the predominant tumor volume, (b) rapid growth or tissue hypoxia with subsequent necrosis (cystic degeneration), (c) cystic metastatic lesions to other organs or omentum, or (d) gross disease treated with appropriate effective therapy in which subsequent cystic lesions develop due to tumor regression [8]. The mechanisms underlying the cystic growth pattern of this neoplasm were not evident on histologic examination. The initial protracted clinical course was suggestive of slow tumor growth; however, cystic degeneration associated with poor vascularization and tissue hypoxia could not be excluded. Interestingly, on computed tomography at time of recheck examination, suspected metastatic lesions also had a cystic structure, implicating a cystic growth pattern in the pathobiology of this mass.

Applying the modified National Institutes of Health (NIH) consensus criteria for defining postsurgical risk of recurrence in humans with GIST, this patient was classified as “high-risk” due to large tumor diameter and mitotic count (> 10/50hpf), and adjuvant tyrosine kinase inhibitor therapy was recommended [9]. Mitotic count has been identified as a prognostic indicator for canine GIST; however, based on the NIH consensus criteria most canine GIST would be classified as “high risk” due to mitotic count alone, and further work to establish risk categories specific for canine patients is recommended [1, 10].

Although imatinib therapy remains the mainstay of treatment of high-risk GISTs in people and has been reported with success in individual dogs, hepatotoxicity is reported in dogs even at low doses which precludes its routine administration [11–13]. Sunitinib is another multi-targeted receptor tyrosine kinase inhibitor that has shown benefit in people who progress despite imatinib therapy [14]. Imatinib, sunitinib and toceranib share affinity for the tyrosine-protein kinase (KIT) receptor, which is the pivotal molecular target of therapy in GIST. As such, toceranib is considered a viable alternative in dogs with similar molecular targets to imatinib and has shown promise in the treatment of GIST in dogs [10, 15].

Despite the NIH “high-risk” designation in 85% of human patients with cystic GIST in one report, the 5-year recurrence rate was only 6.6% [5]. In that same study, a control group of patients with solid GISTs showed a 5-year recurrence rate of 33.9%, significantly higher than those with cystic GISTs [5]. The authors concluded that cystic GISTs may display a relatively indolent behavior and have a favorable prognosis in humans [5]. It is unclear if this advantage is also seen in canine patients.

Gain of function mutations in *c-kit* exon 11 have recently been identified in up to 35% canine GISTs by PCR, and 74% by RT-PCR [16, 17]. The juxtamembrane domain of exon 11 is highly conserved between humans and dogs, being 100% homologous between species [3]. In humans, mutations in *c-kit* are reported in 80 – 85% of GISTs, though a recent publication of cystic GISTs identified *c-kit* mutation in only 45% of patients [4, 5]. It is possible that *c-kit* mutations are less common in human cystic GISTs, though larger studies are required to confirm this finding.

Human patients with mutations of *c-kit* exon 11 appear to have improved response to imatinib therapy, with poorer responses reported for mutations of *c-kit* exon 9 or 17 [18]. However, up to 45% of human wild-type GISTs may respond to imatinib therapy, hence even without identified *c-kit* mutation tyrosine kinase inhibitor therapy may be of benefit [19]. It remains unclear whether genotype analysis and assessment of *c-kit* mutation holds prognostic significance in canine GIST. This patient did not have genotyping performed, as it would not have changed the treatment recommendation or prognostic information based on current veterinary literature.

In conclusion, GIST should be considered as a differential diagnosis in canine patients identified with predominantly cystic lesions within abdominal cavity. The prognosis for these lesions may be improved in comparison to solid GIST lesions based on human literature, though the significance in canine patients remains unclear.

## Data Availability

Data sharing is not considered applicable to this publication, as no datasets were generated or analysed in the creation of this case report.

## Abbreviations

GIST: Gastrointestinal stromal tumor
NIMT: Nonangiogenic, nonlymphogenic intestinal mesenchymal tumor
IHC: Immunohistochemistry
CD117: Cluster of differentiation 117
DOG-1: Discovered-on-GIST-1
NIH: National Institutes of Health
KIT: Tyrosine-protein kinase
PCR: Polymerase chaine reaction
RT-PCR: Reverse transcriptase polymerase chain reaction

## Footnotes

1 IDEXX ProCyte Dx® Hematology Analyzer

2 IDEXX Catalyst Dx® Chemistry Analyzer

3 IDEXX Catalyst One Chemistry Analyser

4 IDEXX Coag Dx Analyser

5 EndoGIA 60mm, TA60mm Autosuture, Medtronic, Auckland

6 Palladia®; Zoetis, Madison, NJ, USA
